# The Use of Plasmapheresis in a Severe Case of Amiodarone-Induced Thyrotoxicosis

**DOI:** 10.1210/jcemcr/luad123

**Published:** 2023-11-03

**Authors:** Tina Moazezi, Chung-Kay Koh

**Affiliations:** Departments of Internal Medicine and Endocrinology, Advocate Lutheran General Hospital, Park Ridge, IL 60068, USA; Departments of Internal Medicine and Endocrinology, Advocate Lutheran General Hospital, Park Ridge, IL 60068, USA

**Keywords:** amiodarone, thyrotoxicosis, plasmapheresis, thyroidectomy

## Abstract

Amiodarone-induced thyrotoxicosis (AIT) can be difficult to treat since amiodarone's long half-life leads to a persistent effect on thyroid function. We present a case of a 74-year-old male with severe AIT who presented with altered mentation and ultimately required intubation and intensive care for management of thyroid storm. Standard medical therapy for treatment of thyroid storm was initiated immediately, but the patient remained unresponsive with worsening biochemical parameters with increasing total T3 levels and sustained elevated levels of free T4 after 5 days of medical management. Due to the lack of a clinical and biochemical response to conventional medical therapy, the patient was started on plasmapheresis and underwent a total of 7 cycles of plasmapheresis over a period of 10 days. He significantly improved with plasmapheresis and was successfully bridged to a total thyroidectomy, which was completed without complications.

## Introduction

Thyroid storm is a serious condition marked by a hypermetabolic state due to excess thyroid hormone. Recognition of thyroid storm requires admission to an intensive care unit for supportive care and close clinical monitoring. When thyroid storm is initially recognized, a multidrug approach is utilized to inhibit thyroid hormone synthesis and secretion and to block the peripheral action of thyroid hormone [1]. In severe cases in which conventional therapy is not effective or contraindicated, or clearance of hormone is necessary for survival, plasmapheresis can be used to achieve clinical stability [2]. Plasmapheresis is effective in removing pathologic substances from the plasma in numerous previously reported hematologic, neurologic, and renal disorders [3]. Its role is less established in thyroid storm due to the availability of medical therapy for treatment and the rarity of the condition [4]. The first report of plasmapheresis for treatment of thyroid storm was published in 1970 [5]. Since then, there have only been case reports and retrospective studies published on the subject, making the available data sparse [4]. In 2019, the American Society of Apheresis changed the use of plasmapheresis in thyroid storm from a category III indication, in which its role should be determined on a case-by-case basis, to a category II indication, in which it was accepted as a second-line therapy either as a stand-alone treatment or in conjunction with other treatments [6]. In this case we present a patient with severe amiodarone-induced thyrotoxicosis who required plasmapheresis as a bridge to total thyroidectomy.

## Case Presentation

A 74-year-old male with a past medical history of type 2 diabetes mellitus, hypertension, hyperlipidemia, anxiety, depression, chronic kidney disease stage IIIa, atrial fibrillation on apixaban and amiodarone, coronary artery disease with history of bypass surgery, and a recent diagnosis of hyperthyroidism on methimazole presented to the emergency department from his rehabilitation facility with altered mentation. The patient was unable to provide a meaningful history despite being functional and independent at baseline.

The patient had recently been admitted for COVID-19 infection and was discharged to a rehabilitation facility 2 days prior. During his previous hospitalization, he was diagnosed with new-onset hyperthyroidism with a thyroid-stimulating hormone level of <.01 mcUnits/mL (<.01 mIU/L) and free T4 level of 3.22 ng/dL (41.45 pmol/L), and he was started on methimazole 10 mg twice daily. He was found to have an elevated C-reactive protein (CRP) of 4.4 mg/dL (419.05 nmol/L) on admission (erythrocyte sedimentation rate was not checked), raising the possibility of subacute thyroiditis in the context of COVID-19 infection. Furthermore, records showed that the patient had been taking amiodarone for the previous 4 years for atrial fibrillation, and his thyroid function had been evaluated approximately 3 months prior. At that time, his thyroid function testing was normal with a thyroid-stimulating hormone level of 1.82 mcUnits/mL (1.82 mIU/L), free T4 level of 1.62 ng/dL (20.85 pmol/L), and free T3 level of 3.07 pg/mL (4.72 pmol/L).

In the emergency department, the patient was afebrile with a blood pressure of 137/60, heart rate of 103 beats per minute, respiratory rate of 34 breaths per minute, and oxygen saturation of 93% on room air. Physical examination was notable for the patient being confused, restless, combative, intermittently responsive to commands, and alert and oriented only to self. The patient had an enlarged thyroid but lacked physical stigmata of Grave's disease, such as thyroid eye disease or thyroid bruit. Inflammatory markers including erythrocyte sedimentation rate and CRP were not checked during the hospitalization, and the patient's encephalopathy upon arrival to our facility prohibited assessment of thyroid tenderness and pain to assess for subacute thyroiditis from prior COVID-19 infection.

On the second day of admission, the patient was noted to have worsening mental status with minimal responsiveness, tachycardia with electrocardiogram demonstrating atrial fibrillation with rapid ventricular response, increased work of breathing requiring escalation of oxygen therapy, and hypotension requiring initiation of vasopressors. The patient was promptly transferred to the medical intensive care unit and intubated. Thyroid function testing revealed an undetectable thyroid-stimulating hormone of <.008 mcUnits/mL (<.008 mIU/L) and profoundly elevated free T4 of >8.0 ng/dL (>102.98 pmol/L), free triiodothyronine of 18.5 pg/mL (28.4 pmol/L), and total T3 of 3.42 ng/mL (5.25 nmol/L). Clinical presentation and laboratory testing was consistent with thyroid storm with a Burch-Wartofsky score of 85. The Burch-Wartofsky point scale predicts the likelihood of thyroid storm by assigning a numerical score to different findings in thyroid storm; a score of 45 or greater is highly suggestive of thyroid storm [1]. It was suspected that our patient had type 2 amiodarone-induced thyrotoxicosis based on the clinical findings and antibody testing, and amiodarone was discontinued. His antithyroglobulin level was 1.0 IU/mL (1.0 kIU/L), thyroid peroxidase antibody was <28 Units/mL (<28 kIU/L), and TSH receptor antibody was <.90 IU/L (< .90 mIU/mL). Thyroid ultrasound revealed an enlarged, diffusely heterogeneous thyroid gland with a left thyroid lobe nodule measuring 1.2 cm, not meeting size criteria for fine-needle aspiration biopsy. A thyroid uptake scan was not performed.

## Treatment

The patient was started on intravenous hydrocortisone 100 mg every 8 hours, enteral propylthiouracil 200 mg every 4 hours, enteral propranolol 60 mg every 4 hours, enteral saturated solution of potassium iodide 300 mg every 6 hours, and enteral cholestyramine 4 g every 6 hours. Total T3 levels were monitored every 8 hours, and free T4 levels were monitored daily. Despite being on maximal medical therapy for 5 days, the patient's total T3 level increased from 3.42 ng/mL (5.25 nmol/L) on initial presentation to 4.80 ng/mL (7.37 nmol/L) on day 5 of treatment, and the patient's free T4 level remained above 8.0 ng/dL (>102.98 pmol/L). The patient remained unresponsive when sedation was weaned, and he continued to spike intermittent fevers.

**Figure 1.**
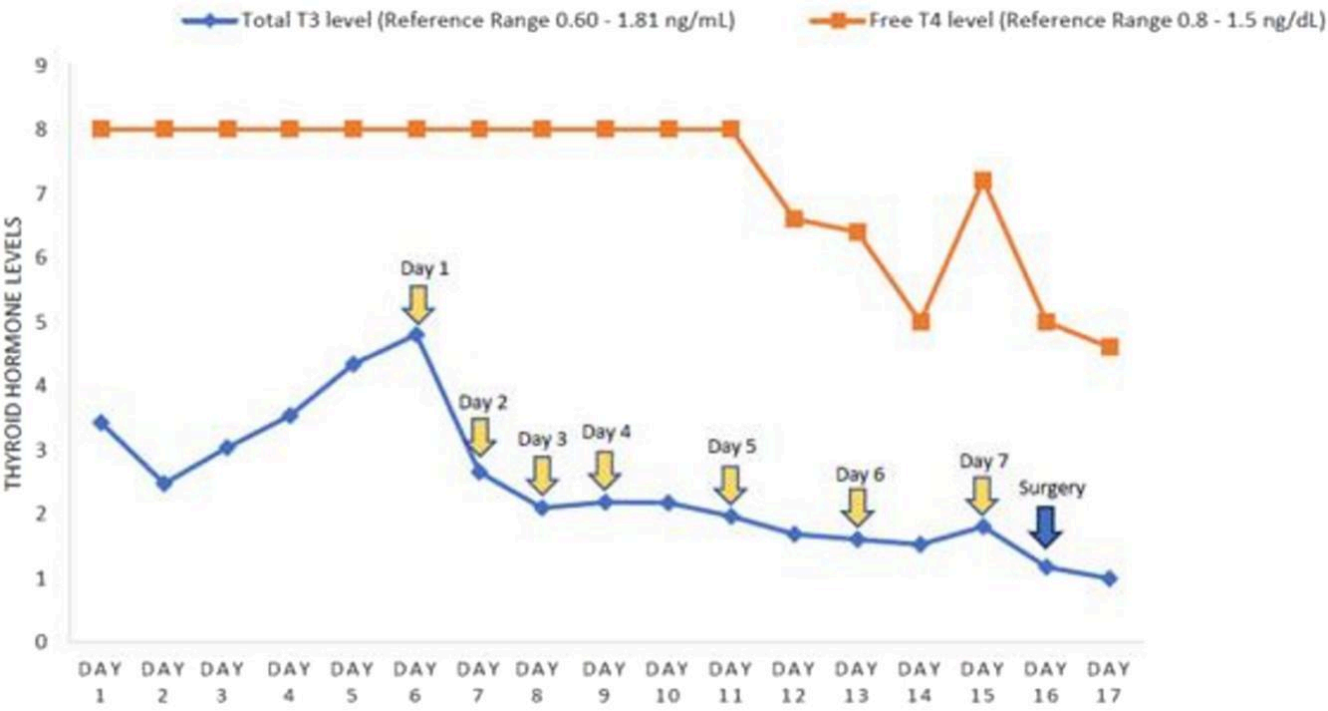
Trend of thyroid hormone levels in relation to timing of therapeutic plasma exchange and eventual thyroidectomy. Free T4 levels through day 11 were above reference range (>8.0 ng/dL). Each cycle of plasmapheresis is denoted by a yellow arrow. Total thyroidectomy is denoted by the blue arrow.

The decision was made to begin plasmapheresis. A double lumen central venous catheter was placed in the right internal jugular vein on the seventh day of hospitalization. Fresh frozen plasma with albumin was utilized as the replacement fluid. The patient was maintained on subcutaneous enoxaparin and sequential compression devices for thromboembolism prophylaxis before and after line placement. The patient's mental status started to improve immediately after the first session. He started to become more alert and regained the ability to open his eyes, move his extremities, and follow commands. Total T3 level improved significantly to 2.65 ng/mL (4.07 nmol/L) after 1 session of plasmapheresis. The patient underwent daily plasmapheresis for the next 4 days (Fig. 1). His mental status showed substantial improvement immediately following each session of plasmapheresis with a gradual decline in mentation throughout each day thereafter.

Plasmapheresis was utilized as a bridging therapy to surgery. The patient had a total of 7 cycles of plasmapheresis over a period of 10 days to remove amiodarone and its active metabolite as well as both free and protein-bound thyroid hormones. This resulted in clinical and biochemical improvement, allowing for a total thyroidectomy to be performed safely on day 16 of admission. Postoperatively, the patient continued hydrocortisone and propranolol to decrease T4 to T3 conversion. On postoperative day 5, the free T4 level was 1.0 ng/dL (12.9 pmol/L), and the patient was started on levothyroxine 100 mcg daily. Hydrocortisone was slowly weaned, and the patient received his final dose on postoperative day 7. A morning cortisol level was checked on postoperative day 9 after being off hydrocortisone for over 24 hours and demonstrated a robust adrenal response at 16.2 mcg/dL (446.96 nmol/L).

## Outcome and Follow-up

As a result of prolonged mechanical ventilation, the patient had placement of a tracheostomy. His mental status improved back to baseline. He was following all commands, was attempting to verbalize, and was alert and oriented to person, place, time, and event. He was finally discharged to his rehabilitation facility on postoperative day 11 in stable condition. The patient was lost to long-term follow-up thereafter.

## Discussion

Our patient's case was unique in that there were 2 potential predisposing factors to the development of thyrotoxicosis: subacute thyroiditis in the setting of COVID-19 infection and amiodarone-induced thyrotoxicosis in the context of long-term amiodarone use. Inflammatory markers were not checked during our patient's hospitalization, but he was noted to have an elevated CRP level 12 days prior at the outside hospital facility. With regards to amiodarone-induced thyrotoxicosis (AIT), specifically type II AIT was suspected given the patient's lack of initial response to antithyroid drugs, lack of clinical findings of thyroid eye disease or thyroid bruit, and negative antibody testing, but a mixed picture of both type I and type II AIT was also possible.

Plasmapheresis can be used in medically refractory cases of thyrotoxicosis to restore hemodynamic and clinical stability [2]. The largest retrospective case series to date involving 46 patients with thyrotoxicosis showed a statistically significant reduction in both free T4 and free T3 levels after a median of 4 sessions of plasmapheresis; this included 40 cases of Graves’ disease, 4 cases of AIT, and 2 cases of toxic nodular goiter [6]. Our case also demonstrated a notable reduction in total T3 level after just 1 session of plasmapheresis with a considerable reduction in free T4 level after multiple cycles of plasmapheresis.

While plasmapheresis is not considered conventional first-line therapy for severe thyrotoxicosis, its effectiveness has been well documented in the literature in certain hematologic, neurologic, and renal disorders, such as thrombotic thrombocytopenic purpura, Guillain-Barre syndrome, and antineutrophilic cytoplasmic antibody-associated vasculitis. The use of apheresis in these conditions carries a category I indication and grade 1A recommendation by the American Society for Apheresis, providing a strong recommendation for plasmapheresis as first-line therapy based on high-quality evidence [7]. The role of plasmapheresis in thyroid storm is less established due to the availability of effective multidrug therapy for treatment and given how rare medically refractory thyroid storm is. As a result, only case reports, case series, and retrospective studies demonstrating the efficacy of plasmapheresis in thyroid storm have been published [8].

There are multiple reasons why plasmapheresis is favored in thyroid storm over other purification methods such as hemodialysis or hemofiltration. These include the ability to remove substances with a long half-life as well as substances with a large molecular weight. During plasmapheresis in thyroid storm, thyroid-binding globulin and bound thyroid hormones are removed with the plasma, and it is therefore effective in removing both free and protein-bound thyroid hormones [9]. Plasmapheresis is particularly effective in lowering plasma concentrations of amiodarone in cases of AIT due to the long half-life of amiodarone and the tight binding of amiodarone and its active metabolite to plasma proteins [10]. Serum amiodarone levels were not monitored during our patient's hospitalization, and previous case reports of AIT treated with plasmapheresis do not trend serum amiodarone levels with each cycle of plasmapheresis. In our case, total T3 and free T4 levels were closely monitored to determine the patient's response to plasma exchange and the timing of surgery. Removal of amiodarone and its active metabolite with plasma exchange leads to quicker improvement of thyroid function, allowing for lifesaving thyroidectomy to take place sooner.

The efficacy of plasmapheresis in thyroid storm has been documented in numerous case reports and case series. In our case, plasmapheresis led to decreases in total T3 concentrations of roughly 45% after 1 session and roughly 76% reduction with 7 sessions of plasmapheresis by the time of thyroidectomy. In contrast, free T4 levels remained elevated above reference range until the day after the fifth session of plasmapheresis (unable to calculate percentage reduction due to exact lab value of free T4 being above reference range and therefore unknown until day 12 of hospital admission). The exact number of plasmapheresis sessions needed prior to thyroidectomy varies on a case-by-case basis based on response to therapy. In case reports of AIT requiring plasmapheresis, patients generally achieved a normal or near normal free T3 level before total thyroidectomy [9]. Ultimately, while plasmapheresis is not conventionally used as part of routine treatment for thyroid storm, endocrinologists should keep this tool at the forefront of their management strategies to deliver quicker treatment to patients and prevent complications of thyroid storm. Nonetheless, more research is needed to delineate concrete guidelines and specific protocols for its use in thyroid storm.

## Learning Points

- Plasmapheresis should be considered in severe cases of thyrotoxicosis when patients do not respond to standard multidrug medical therapy.
- The long half-life of amiodarone can lead to amiodarone-induced thyrotoxicosis, and plasmapheresis can be beneficial in reducing plasma concentrations of amiodarone as well as circulating thyroid hormones safely and quickly.
- Plasmapheresis can be used to decrease thyroid hormone levels in severe cases of thyrotoxicosis to safely bridge patients to definitive treatment with thyroidectomy.

## Contributors

C.K. was involved in the care of this patient and assisted with manuscript review, and T.M. wrote the manuscript. All authors reviewed and approved the final draft.

## Data Availability

Data sharing is not applicable to this article as no datasets were generated or analyzed during the current study.
